# Actinobacterial Rare Biospheres and Dark Matter Revealed in Habitats of the Chilean Atacama Desert

**DOI:** 10.1038/s41598-017-08937-4

**Published:** 2017-08-21

**Authors:** Hamidah Idris, Michael Goodfellow, Roy Sanderson, Juan A. Asenjo, Alan T. Bull

**Affiliations:** 1 School of Biology, Ridley Building, Newcastle University, Newcastle upon Tyne, NE1 7RU United Kingdom; 2 Centre for Biotechnology and Bioengineering (CeBiB), Department of Chemical Engineering and Biotechnology, University of Chile, Beauchef 851, Santiago, Chile; 3 School of Biosciences, University of Kent, Canterbury, CT2 7NJ Kent, United Kingdom

## Abstract

The Atacama Desert is the most extreme non-polar biome on Earth, the core region of which is considered to represent the dry limit for life and to be an analogue for Martian soils. This study focused on actinobacteria because they are keystone species in terrestrial ecosystems and are acknowledged as an unrivalled source of bioactive compounds. Metagenomic analyses of hyper-arid and extreme hyper-arid soils in this desert revealed a remarkable degree of actinobacterial ‘dark matter’, evidenced by a detected increase of 34% in families against those that are validly published. Rank-abundance analyses indicated that these soils were high-diversity habitats and that the great majority of designated ‘rare’ genera (up to 60% of all phylotypes) were always rare. These studies have enabled a core actinobacterial microbiome common to both habitats to be defined. The great majority of detected taxa have not been recovered by culture dependent methods, neither, with very few exceptions, has their functional ecology been explored. A microbial seed bank of this magnitude has significance not just for Atacama soil ecosystem resilience but represents an enormous untapped resource for biotechnology discovery programmes in an era where resistance to existing antibiotics is rapidly becoming a major threat to global health.

## Introduction

Research on extremophilic and extremotrophic microorganisms has developed rapidly since the mid-1970s as microbiologists recognized that extreme environments were capable of sustaining life. Subsequent delineation of the extremobiosphere has shown it to encompass all of the physico-chemical variables on Earth and that many microorganisms have evolved tolerances to multiple extreme conditions. Our research in this context has shown that members of the phylum *Actinobacteria* ^1^ are found throughout the extremobiosphere^2^, and is based on the rationale of size and diversity of the taxon, its geographic dispersal, and its ecological and biotechnological relevance. We have focused on two extreme environments, the deep-sea^3^ and more recently hyper-arid desert. Over 22% of the terrestrial environment comprises desert of which a third is classified as hyper-arid (ratio of mean annual rainfall to mean annual evaporation (MAR) less than 0.05) or extreme hyper-arid (ratio < 0.002)^4^.

The Atacama Desert located in northern Chile is the most ancient and continuously driest non-polar environment on Earth; apart from its aridity it is unique in its range of habitats, its geology and geochemistry, its elevation and topography, and its radiation intensities^5^. Previous work by our group has enabled cultivable actinobacteria to be recovered from a range of Atacama soils, regoliths and rock surfaces^6, 7^, including ones purporting to represent “the dry limit of microbial life”^8^, and from which have been isolated several new species and natural products possessing sought for bioactivities^9, 10^ and whole genome sequences determined^11, 12^. However, although there are clear indications from us and other groups that actinobacteria are widespread and frequently dominate prokaryotic communities in the Atacama Desert (for example, 75% and 98% in low^13^ and high^14^ elevation soils), this has not been supported by culture-based studies. Consequently the low diversity of actinobacteria observed using selective cultivation techniques^6^ has provided impetus for the present investigation. As Garza and Dutilh^15^ have emphasized metagenomics now is enabling comprehensive surveys of microbial communities in diverse environments that culture-based approaches have not achieved and as high throughput molecular techniques become cheaper and more effective they are being adopted as default tools for such investigations^16^. Roche 454 and Illumina technology platforms have been widely adopted for metagenomic studies, informative comparisons of which have been made on natural microbial communities^17, 18^. Roche GS-FLX sequencing systems have started to be applied to microbial community analyses in the Atacama Desert and have revealed a dominance of actinobacteria in hyper-arid margin soils^13^, and bacterial colonization patterns along longitudinal moisture gradients^19^. As a first step in such an approach specifically to actinobacterial diversity in the Atacama Desert we have carried out community profiling based on 454 pyrosequencing. Our choice of this particular platform in part was based on its high classification efficiency^17^, and evidence that deep pyrosequencing of 16S tags is well-suited for distinguishing site specific similarities and differences among rare bacteria^20^.

The principle objectives of this study were to determine how diverse actinobacterial communities are in the Atacama Desert and how big a resource this presents for biotechnology exploitation, and to dissect such diversity in terms of actinobacterial rare and previously undetected dark matter^21, 22^. To this end, we have applied R H Whittaker’s classic diversity indices^23^ to actinobacterial diversity of a hyper-arid—extreme hyper-arid landscape at five distinct habitats covering an altitude range of approximately 900–2500 m absl. Based on trial experiments analyses were restricted to generic and family ranks and we have not attempted correlations of local diversity (*sensu* α-diversity^24^) with environmental factors. In addition, we hypothesized that this desert landscape would be propitious for exploring the rare biosphere concept, and the notion of actinobacterial signatures defining the habitats. We opine that the results obtained reinforce the view that rather than being devoid of life the Atacama Desert is a microbial treasure trove in the search for the next generation of bioactive natural products.

The purpose of this paper is to report the remarkably high diversity of actinobacteria in the Atacama Desert and highlight its exceptional significance for biodiscovery campaigns. Metagenome sequencing has revealed a strong correlation between global geographic distance and biome type and the biosynthetic diversity residing in soils^25^, a conclusion that strongly resonates with our decision to explore the unique and poorly studied microbiomes of the Atacama Desert.

## Results

**Figure 1:**
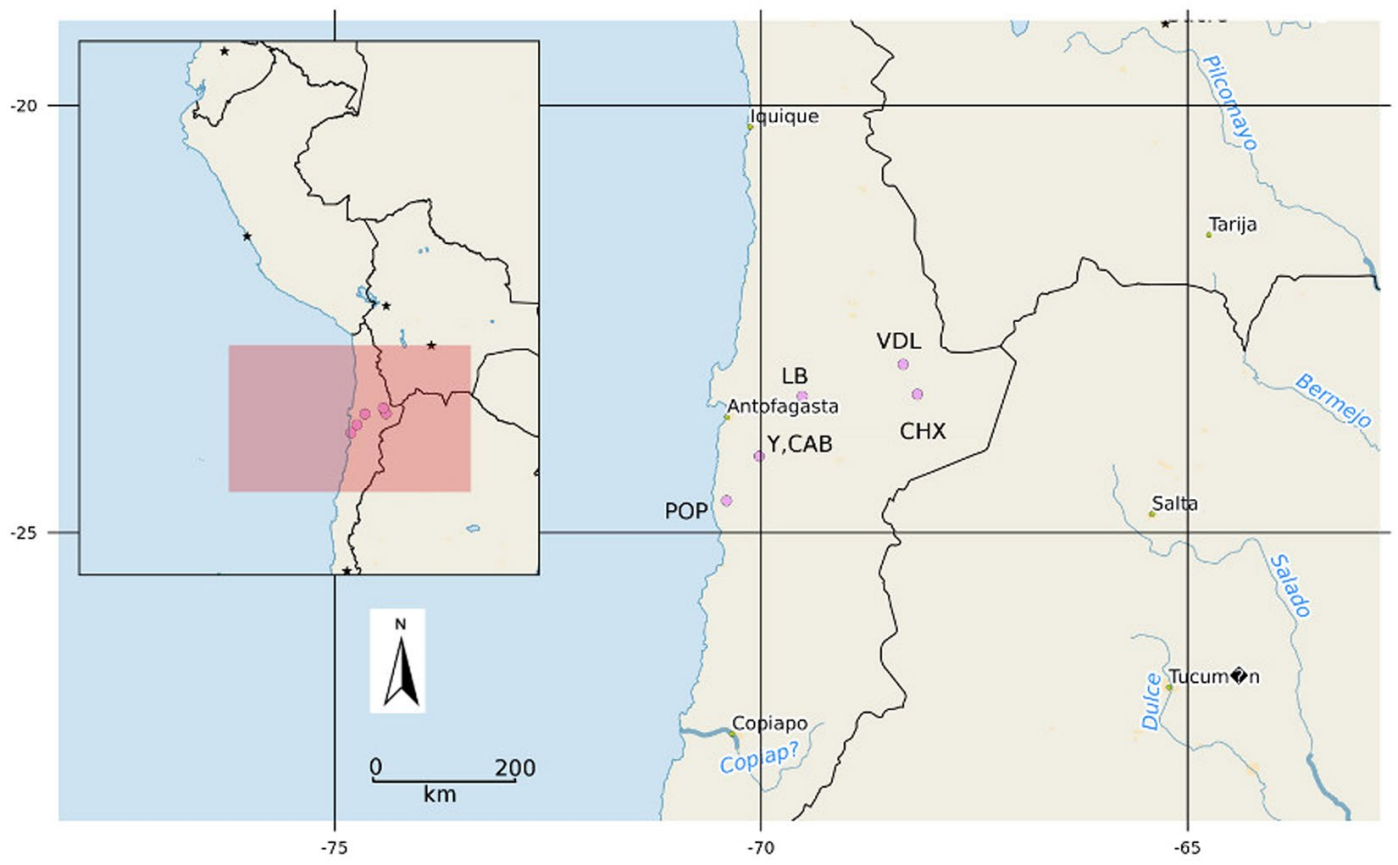
Location of Atacama Desert research in northern Chile. Site codes: Yungay (Y); Cerros Aguas Blancas (CAB); Lomas Bayas (LB); Cerro Paranal (POP); Salar de Atacama (CHX); Cordillera de la Sal (VDL). Map created by RAS from OpenStreetmap cartography (licensed under CC BY-SA http://www.openstreetmap.org/copyright © OpenStreetMap contributors) using QGIS version 2.14 (Open Source Geospatial Foundation http://qgis.org/en/site).

Over 90,000 sequence reads were obtained from the 12 sites (Fig. 1) representing 67 actinobacterial families, 16% of which could not be assigned to validly published taxa and therefore, are regarded as putatively novel candidate families (see Table S1). The large majority of identified families belonged to the class *Actinobacteria* ^26^ but in addition a few representatives of rare deep lineage actinobacteria belonging to the classes *Acidimicrobia* ^27^ and *Nitriliruptoria* ^28^ were detected. The total detected diversity at the generic rank numbered 297 only 60% of which were assigned to validly published taxa.

Pyrosequencing data for microbiota diversities in other world deserts show significant variations in the occurrence of actinobacteria. Thus, sandy Asian deserts such as the Gobi, Gurbantünggütt and Taklamaken are reported to have small (2–18%) actinobacterial communities^29, 30^. In contrast, soils of certain western deserts such as the Atacama and Sonoran are heavily dominated by actinobacterial phylospecies (75–88%^13, 19^). Culture-dependent values obtained for the sites examined in the present study are 50–70% (hyper-arid) and up to 100% in certain extreme hyper-arid samples (Valle de la Luna^6^). Some acuity should be exercised in reviewing such measures of dominance, or otherwise, as actinobacterial counts are influenced significantly by the selective procedures used for their isolation^6^. The taxonomic megadiversity of actinobacteria in the Atacama Desert landscape as revealed for the first time in this study argues strongly for their successful adaptation to extreme resource-depleted conditions.

### Rarefaction analysis

**Figure 2:**
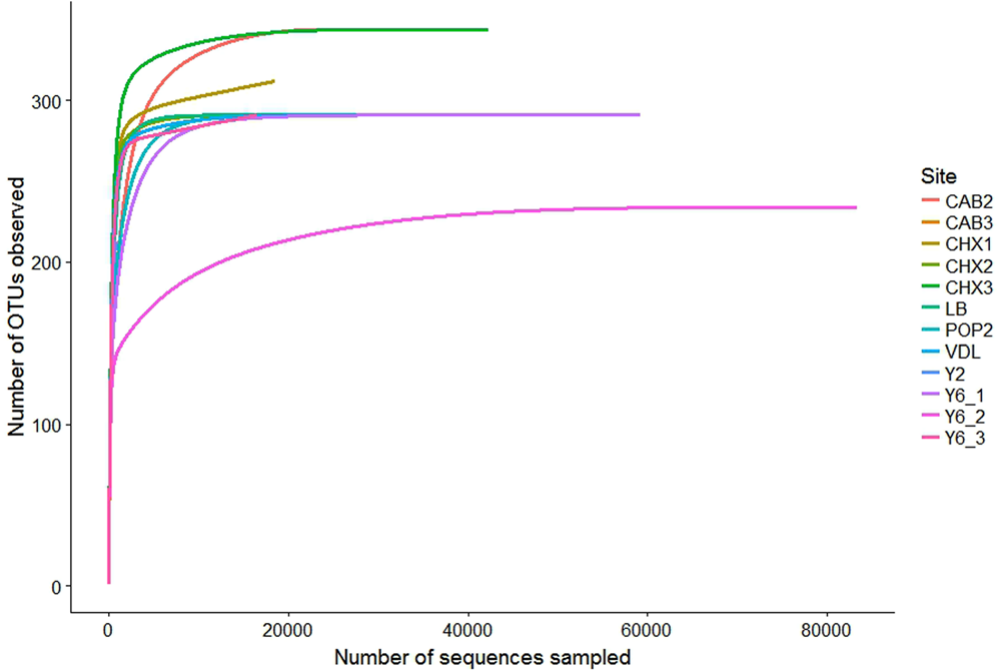
Rarefaction analysis of the extreme hyper-arid and hyper-arid OTUs at 94.5–97% phylogenetic similarity.

Construction of rarefaction curves enabled us to compare genus richness and gauge the extent to which total diversity had been recovered at each of the Atacama sites (Fig. 2). In most cases the major extent of the actinobacterial community diversities had been captured by our sequencing campaign at the 1500-sequence level but in one case (CAB3) the sequence level of 1054 obtained fell well below this value and likely compromised subsequent analyses of this site. Most of the curves closely approached asymptotes; a few others remained curvilinear indicating that further actinobacterial diversity remained to be sequenced. Thus it is important to recognize that taxon counts are strictly comparable only when richness is asymptotic, a situation pertaining to our observations. The efficiencies of sequence capture in a few of the sites examined (Yungay Y6.1, Y6.2, Y6.3; Chaxa Laguna CHX1, CHX2) were not influenced by sampling depth.

Application of Good’s library coverage index indicated that genotype recovery reached 90% to 97% at all sites. Table 1 presents total OTUs (total observed validly published genera, plus unidentified genera) and α-diversity indices. Only a small decrease (*ca*. 7% average) was found in the observed OTUs recovered from surface soil samples as a function of increased aridity.

**Table 1:** Observed OTUs and α-diversity indices.

Sampling site and depth (cm)	Observed OTUs (Genera)	Chao1 Richness	Shannon Index (*H*)	Simpson Diversity (1/*D)*
**1. Extreme hyper-arid locations** ^1^				
**Yungay**				
Y6_1 (2)	186	316	4.01	19.2
Y6_2 (30)	87	134	2.78	7.9
Y6_3 (100)	159	222	3.87	19.6
CAB2 (2)	175	331	3.73	16.9
CAB3 (2)	63	110	3.40	20.8
**Lomas Bayas**				
LB (1)	162	264	3.54	10.1
**Cordillera de la Sal**				
VDL (2)	185	242	4.29	27.8
**2. Hyper-arid locations** ^2^				
**Laguna Chaxa**				
CHX1 (2)	222	306	4.48	43.5
CHX2 (30)	208	295	4.38	29.4
CHX3^3^ (2)	171	247	3.73	14.7
**Cerro Paranal**				
POP2 (2)	157	228	3.40	9.1
**Yungay Oasis**				
Y2^4^ (2)	89	137	3.21	15.4

^1^MAR 0.002, ^2^MAR 0.009, ^3^Partly colonized by cyanobacteria, ^4^Tamarisk grove.

Comparable data for other desert environments are largely lacking but increased salinity was not found to be a major determinant of species richness in saline lakes of the Monegros Desert in NE Spain^31^. Neither did we observe a major change in overall actinobacterial ecological diversity (*H*, 1-*D*) between extreme hyper-arid and hyper-arid locations, a result indicating that they are dominated by relatively few abundant taxa. Chao 1 predicted richness was high at both types of location exceeding the observed OTU numbers by 63% (extreme hyper-arid) and 41% (hyper-arid) and providing further evidence of diversity yet to be sequenced. It should be noted that non-parametric richness estimators such as Chao 1 predict counts of the number of taxa (in this case genera and families) present at a site but cannot be used to compare the genetic diversity between sites^32^. The reciprocal of the Simpson index (1/D) is sensitive to the degree of dominance in a community^33^; increases in the index indicate increases in diversity. Values below about 50 have been taken to indicate typical dominance profiles^34^. With the exception of the CHX1 value which appears to lie near the boundary between dominant and uniform community profiles, all other Atacama Desert communities surveyed here show dominance (see Taxonomic diversity below).

### Rank-abundance distributions

**Figure 3:**
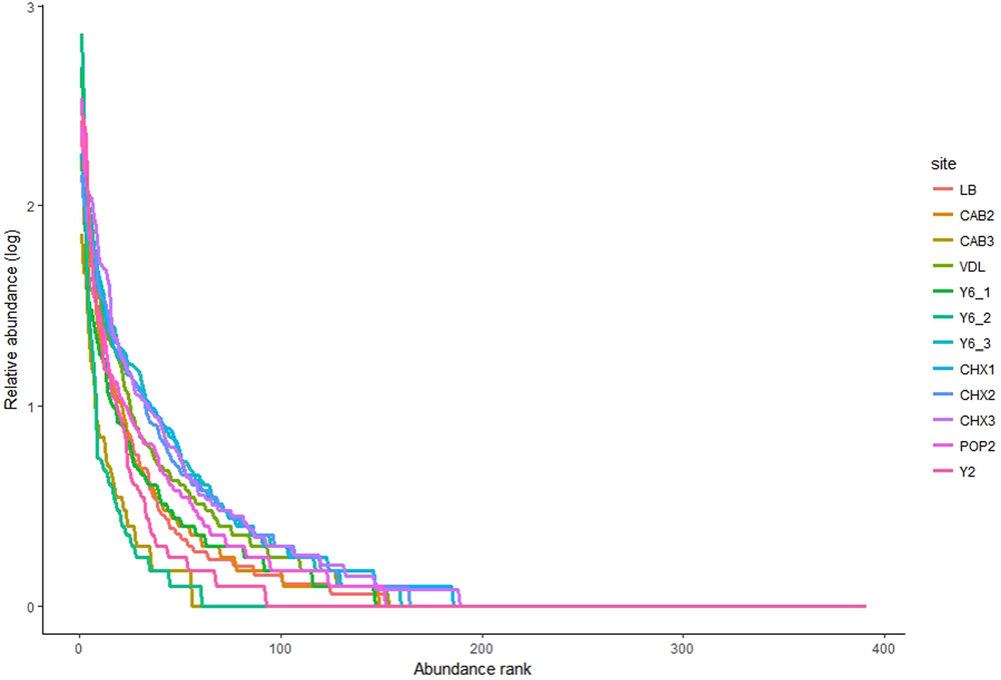
Rank-abundance curves.

For the purpose of defining rank-abundances in Atacama soils we have used a relative cut-off of 0.1%^35, 36^ below which threshold a rare actinobacterial biosphere can be defined; and an arbitrary threshold of 10% to define abundant taxa^36–38^. Rank-abundance curves for all of the research sites are shown in Fig. 3.

Salient points arising from these analyses: (1) the majority of curves are indicative of high-diversity environments, i.e. shallow curve and a long tail represent a rare biosphere; (2) the great majority of rare generic OTUs in the Atacama habitats were always rare (>95%) and were not found as abundant at any of the environments examined; (3) the proportions of abundant and rare actinobacterial taxa were similar at extreme hyper-arid and hyper-arid sites (e.g. 9–17% and 9–15% abundant vs. 46–62% and 48–61% rare genera respectively; (4) on the basis of a small number of comparisons, the composition of the abundant communities in surface, sub-surface and deep samples was similar (e.g., Cerros Aguas Blancas sites at 2, 30 and 100 cm were dominated by *Blastococcus, Verrucosispora* and unidentified OTUs FJ479147_g, HQ674860_g and HQ910322_g; while Laguna Chaxa sites at 2 and 30 cm were dominated by *Arthrobacter, Blastococcus* and unidentified OTU HQ910322_g); (5) the taxonomic constancy of abundant OTUs observed as a function of soil depth argues for long term habitat stability and minimal atmospheric aerosol dispersal of Atacama microbiota, a conclusion supported by aerosol optical depth measurements made in our research landscape^39^; and (6) at genera and family ranks a very much higher number of unassigned OTUs were evident in the rare biosphere confirming that the Atacama landscape represents a vast reservoir of microbial dark matter.

### Taxonomic diversity

**Figure 4:**
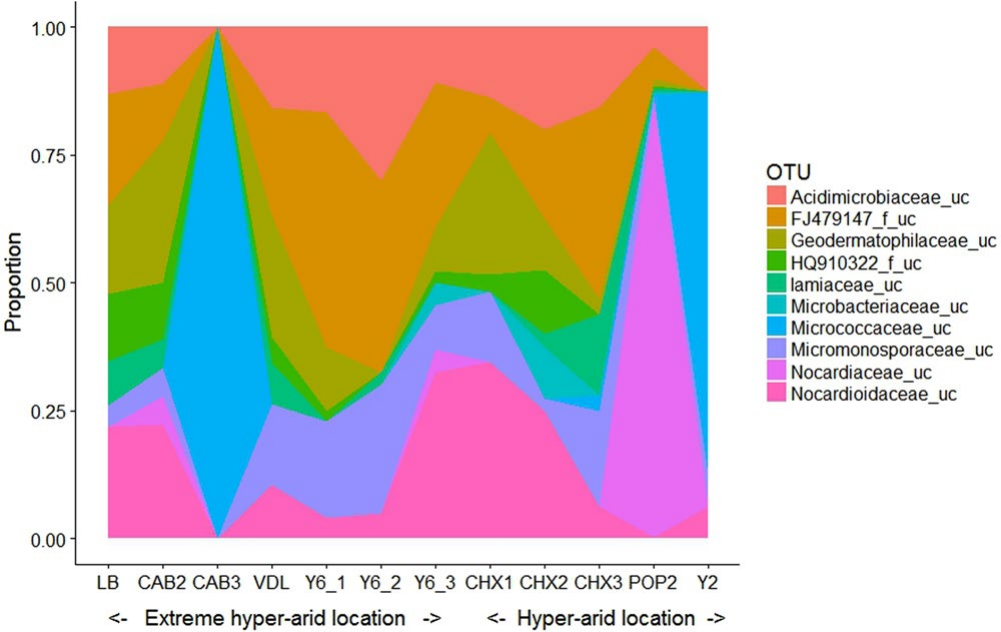
Relative abundance of top 10 most frequently detected actinobacterial families in Atacama Desert locations.

The profiles of actinobacterial family lineages throughout the landscape locations were similar and dominated by members of the families *Acidimicrobiaceae, Geodermatophilaceae, Iamiaceae, Microbacteriaceae, Micrococcaceae, Micromonosporaceae, Nocardiaceae* and *Nocardioidaceae* and two unidentified taxa, FJ479147_f and HQ910322_f. The relative abundance of the top ten families (Fig. 4) shows clearly how community structure was affected by soil depth (Y6 and CHX series) and how at two sites (Y2, CAB3) the communities were dominated by OTUs belonging to the family *Micrococcaceae*. At this stage we have insufficient data to interpret the shifts in the structures of the latter communities except to note that site Y2 was partially vegetated, and that variations in community structure observed at sites Y6.1, CAB2 and CAB3 reinforce the importance of recognising spatial and temporal factors in habitat sampling. The majority of the most abundant identified families belong to the orders *Acidimicrobiales, Geodermatophilales, Micrococcinales* and *Micromonosporiales* ^40, 41^, actinobacterial taxa notable for their extremophilic and extremotrophic members^2^. Currently 58 validly published families are recognised in the phylum *Actinobacteria* (www.bacterio.net at 08/07/2016); consequently the additional 23 taxa recorded as ‘families_f’ constitute a massive element of actinobacterial dark matter in the Atacama landscape.

**Figure 5:**
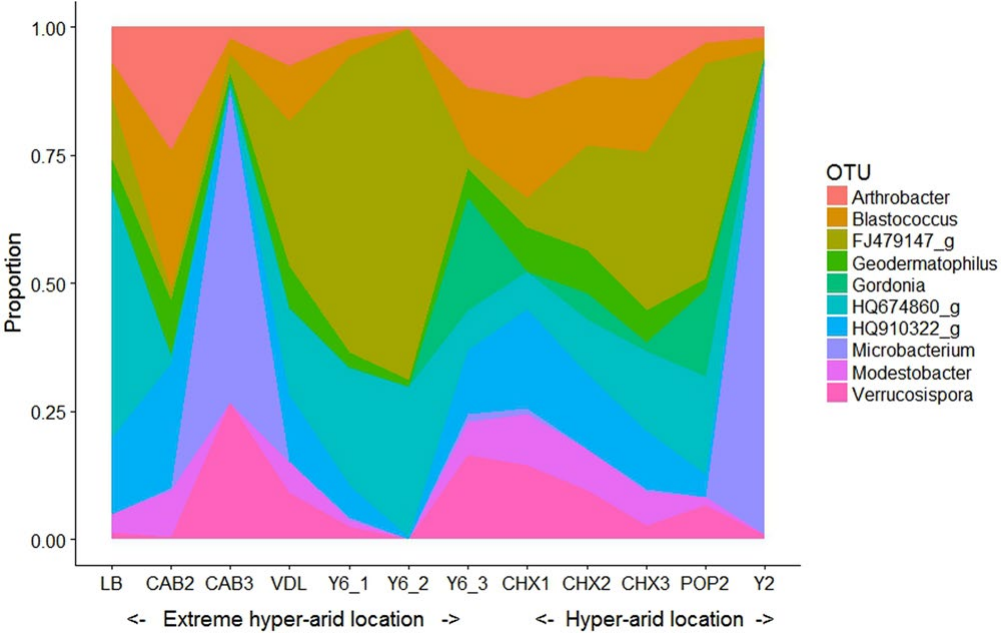
Relative abundance of actinobacterial genera detected in Atacama Desert locations. Relative abundance shown at 2.5% cut off.

The total number of generic OTUs in each sample is too large to render relative abundance diagrams readily decipherable at the 0.1% cut-off level, consequently data presentation is shown for 2.5% cut-off levels in Fig. 5; the full list of the 297 genera is given in Table S2. The genera dominating the various sites were *Arthrobacter, Blastococcus, Geodermatophilus, Gordonia, Microbacterium*, *Modestobacter and Verrucosispora*, and three unidentified putatively novel taxa FJ479147_g, HQ674860_g and HQ910322_g. Although the proportions of these genera varied from site to site, generally they comprised 75% of the community and, predictably, showed high correspondence with family dominance at the same sites. Figure 5 defines the distinctive nature of sites CAB3 and Y2 in which the genus *Microbacterium* comprised 48% and 58% of these communities respectively. The genera *Kocuria, Nocardioides, Sanguibacter* and *Streptomyces* were found to be relatively abundant in both the hyper-arid and extreme hyper-arid core microbiomes (Table 2). The few genera recorded as deep lineage actinobacteria (*Aciditerrimonas, Iamia, Ilumatobacter, and Nitriliruptoraceae_uc*) were mainly detected at very low read levels at certain sites and can be regarded as constituents of the rare actinobacterial biosphere (see below) in the Atacama landscape. Although *Acidimicrobiaceae* was found to be among the dominant families, *Ilumatobacter* was the only member genus detected and in most cases as a constituent of the rare biosphere.

**Table 2:** Most abundant genera of the extreme hyper-arid and hyper-arid core microbiomes.

Extreme hyper arid	Hyper arid
FJ479147_g	FJ479147_g
HQ674860_g	HQ674860_g
*Blastococcus*	*Microbacterium*
HQ910322_g	*Kocuria*
*Arthrobacter*	*Sanguibacter*
*Verrucosispora*	*Verrucosispora*
*Geodermatophilus*	HQ910322_g
*Modestobacter*	*Nocardioides*
*Microbacterium*	*Gordonia*
*Sporichthya*	*Blastococcus*
*Friedmanniella*	*Corynebacterium*
*Streptomyces*	*Arthrobacter*
*Sanguibacter*	*Streptomyces*
*Kocuria*	*Aciditerrimonas*
*Nocardioides*	FJ478790_g

The order of genera indicates their comparative dominance within each microbiome.

### Habitat specificity and co-occurrence

**Figure 6:**
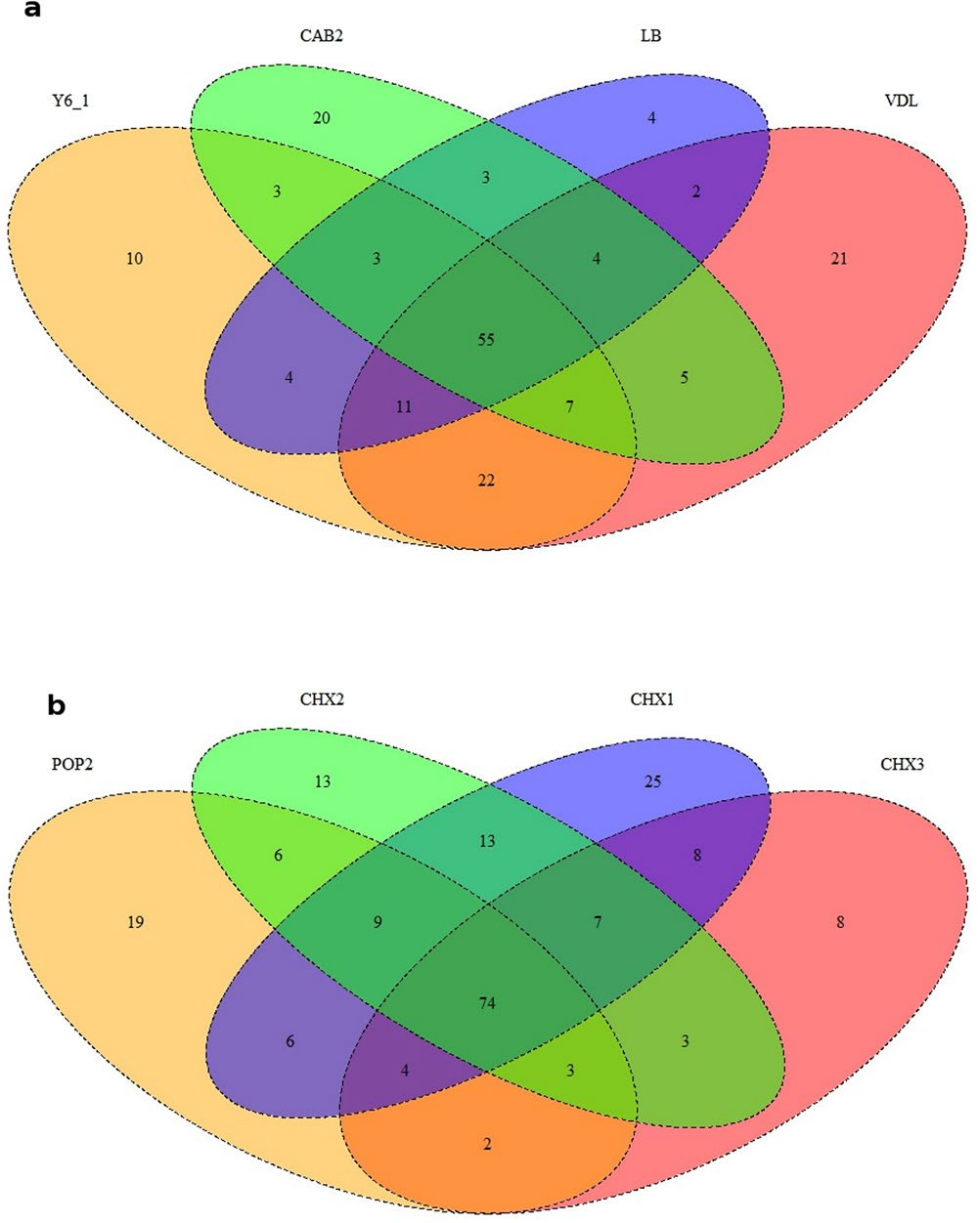
Venn diagrams showing proportions of shared and unique actinobacterial genera at (**a**) extreme hyper-arid and (**b**) hyper-arid sites.

Venn diagram analyses showed that the proportion of surface community genera shared between sites within extreme hyper-arid (32%) and within hyper-arid (37%) locations was comparable (Fig. 6). However, similar analyses highlighted the effect of vegetation on hyper-arid community composition and of soil depth on extreme hyper-arid shared community composition reducing them to 14% and 22%, respectively.

Venn diagrams can provide a ‘reasonable first exploration’ of defining the ‘core microbiome’ of a habitat^38^ and we have adopted this approach to describe organisms shared across the Atacama landscape. Although we have focused solely on the phylum *Actinobacteria* we opine that it is credible to construct a core microbiome based upon this dominant component of the Atacama microbiota. Figure 6 indicates that 55 and 74 respectively of the OTUs were shared in extreme hyper-arid and hyper-arid surface sites. The most abundant OTUs are almost identical in the two biomes (Table 2) suggesting an actinobacterial signature and postulating that they play a key role in ecosystem function within these Atacama soils. However, the genera *Friedmaniella* and *Sporichthya* were abundant in the extreme hyper-arid core microbiome and the genera *Aciditerrimonas* and *Corynebacterium* and the putatively novel taxon TJ478790g in the hyper-arid core microbiome (Table 2).

## Discussion

The results reported here confirm that actinobacteria constitute a major and frequently dominant component of desert soils. Remarkable, however, is the megadiversity of this phylum throughout hyper-arid and extreme hyper-arid habitats in the world’s driest desert such that our metagenomics data set revealed a 16% greater coverage of actinobacteria at the family rank than that currently recognized. Similar actinobacterial dark matter was evident at the generic level where 40% of the captured diversity was not ascribable to validly published genera. Equally remarkable are the high proportions of unidentified (*No-Rank*) and very low abundance OTUs, features that are also found in other extreme and poorly researched ecosystems^42, 43^. Although caution is required in interpreting such metagenomic data^44^ this study provides a sound base from which to explore an astonishing and unpredicted desert microbiota.

An actionbacterial core microbiome dominated by FJ479147_g, HQ 674860_g, HQ910322_g*, Arthrobacter, Blastococcus, Geodermatophilus, Gordonia, Microbacterium* and *Verrucosispora* (a phylogenetic relative of the genus *Micromonospora* ^45^) defined the sampling sites. The genera *Friedmaniella*, *Nocardioides*, *Sanguibacter*, *Sporichthya* and *Streptomyces* were relatively abundant in the extreme hyper-arid core microbiome and the genera *Aciditerramonas* and *Corynebacterium* in the hyper-arid core microbiome. Most of the genera found in these core microbiomes encompassed amycelial actinobacteria classified in the families *Geodermatophilaceae* (*Blastococcus, Geodermatophilus* and *Modestobacter*). *Micrococcaceae* (*Arthrobacter*), *Microbacteriaceae* (*Microbacterium*), *Propionibacteriaceae* (*Friedmaniella*) and *Sanguibacteriaceae* (*Sanguibacter*)^40^. These genera, apart from those classified in the family *Geodermatophilaceae*, are not usually associated with desert habitats though *Arthrobacter* strains have been isolated from Yungay soil^40^ and *Friedmaniella antarctica* and *Sanguibacter antarticus* from Antarctic sea sand^46, 47^.

Exploration of the microbial world has recently been revolutionised by the advent of rapid and inexpensive DNA sequencing technology. In the past assessments of species ‘rarity’ were largely subjective and relied on culture-dependent surveys but now the ability to construct and analyse very large rank-abundance data sets has enabled rare biospheres to be defined with confidence. However, it is important to recognise that truly rare sequences in environmental samples may escape detection due to incomplete sequencing^20^. The actinobacterial rare biosphere determined within the Atacama landscape is the first to be reported for highly arid environments and accords with the few broader microbial surveys of other deserts (e.g. Sonoran Desert^48^). A notable feature of the Atacama rare biosphere is the high frequency of taxonomically unassigned OTUs confirming that this landscape contains a vast reservoir of actinobacterial dark matter. Although the functional ecology of the Atacama’s actinobacteria remains to be researched it provides an immense microbial seed bank whose role in soil ecosystem resilience warrants investigation. This hypothesis is given credence by recent reports that members of rare bacterial taxa became dominant in spring sediment microcosm experiments following exposure to environmental stressors^49^ and are resuscitated in a variety of ecosystems following rewetting of dry soils^50^.

The results of this study commend several future research imperatives among which we highlight the following: (1) develop innovative isolation/cultivation techniques to enable whole organism physiology of desert microorganisms to be determined; (2) evaluate the viability and metabolic activity of desert microbiomes using, for example, microcosms^19, 49^, DNA stable isotope probing which to date has not been utilized in desert ecology, and ribosomal tag pyrosequencing^51^; (3) apply functional genomics to explore ecological traits in desert microorganisms; the genome of *Modestobacter caceresii* recently isolated from the extreme hyper-arid Yungay region of the Atacama Desert contains 110 genes that are associated with stress responses including those involved in heat and cold shock responses, osmotic stress, and carbon starvation^12^; and (4) develop the biotechnological potential of this huge reservoir of desert “biosynthetic dark matter”^52^ access to which has become greatly facilitated by genome and metagenome mining as evidenced by the discovery of new antibacterial/anticancer ansamycins^53^, synthesized by a novel strain, *Streptomyces leeuwenhoekii*, isolated from a hyper-arid Atacama Desert soil^7^.

## Methods

### Sample collection and research landscape

Soil samples (Table 3) were collected from selected locations in the Atacama Desert landscape (Fig. 1) between 2010 and 2011 (ATB, MG), and additional ones in 2004 (ATB), and 2012 (Professor Luis Cáceres, University of Antofagasta). Samples were collected aseptically using implements sterilised in the field with ethanol and contained in sterile polycarbonate bottles. Sampling was made between 11:00 and 16:00 h during which period temperature and relative humidity at the lower elevation sites ranged between 34°–38 °C and 5–20% respectively, and 30°–33 °C and 3–18% respectively at the higher altitudes. Following transport to the UK all samples were stored at 4 °C.

**Table 3:** Sampling locations.

Research location	Sampling site and code	Collection date	Latitude (°S)	Longitude (°W)	Altitude	Sample description
**Yungay**	(1) Tamarisk oasis					
	Y2	11.11.2010	24°04′50.5″	69°55′08.3″	918	Degraded tamarisk leaves and surface nitrate soil
	(2) Cerros Aguas Blancas					
	Y6_1	13.11.2010	24°06′18.4″	70°01′15.4″	1002	Extreme hyper-arid site, fine white soil (surface 2 cm)
	Y6_2	13.11.2010	24°06′18.6″	70°01′15.6′	1002	Sub-surface (30 cm)
	Y6_3	13.11.2010	24°06′18.6″	70°01′15.6′	1002	Sub-surface (100 cm)
	CAB2	30.10.2011	24°05′24.9″	69°58′31.9″	1060	Surface (2 cm)
	CAB3	30.10.2011	24°06′40.3″	70°02′03.5″	1079	Surface (2 cm)
**Lomas Bayas**	LB1	26.10.2012	23°24′27.4″	69°31′03.8″	1500	Extreme hyper-arid, surface soil
**Cerro Paranal**	POP2	30.10.2011	27°75′	68°42′	1945	Coarse sandy hyper-arid, surface soil
**Salar de Atacama**	Laguna Chaxa					
	CHX1	26.10.2012	23°17′33″	68°10′99″	2219	Hyper-arid halite (surface 2 cm)
	CHX2	26.10.2012	23°17′33″	68°10′99″	2219	Sub-surface (30 cm)
	CHX3	26.10.2012	23°17′36″	68°10′83″	2222	Soil colonized by cyanobacteria
**Cordillera de la Sal**	Valle de la Luna					
	VDL	05.10.2004	23°02′	68°20′	2450	Extreme hyper-arid sand

The Yungay area is the extreme hyper arid core of the Atacama Desert and is considered to be the closest analogue of Martian soils on Earth^54^. A large proportion of the area is encrusted with halite and super-rich in nitrates, while certain slopes on the Cerros Aguas Blancas present evidence of water erosion in geological time. Two Yungay sites were sampled: (1) nitrate rich soil from a small tamarisk oasis (Y2); and (2) transect samples from the Cerros Aguas Blancas (CAB series, Y6) WSW of the Cerro Caballo Muerto. The Lomas Bayas region is another extreme hyper-arid environment and a center of copper mining located north east of Antofagasta; samples (LB) were collected at non-mining sites. Sampling was made on the eastern slope of Cerro Paranal adjacent to Route B-710 linking Antofagasta and the coastal village of Paposo. The Salar de Atacama is the largest salt flat in Chile within which the Laguna Chaxa is an area of open water and highly crystalline salt encrusted soils. Samples (CHX) were collected from the halite soils one of which was partially colonized by cyanobacteria. The Valle de la Luna is an extreme hyper-arid area in the Cordillera de la Sal; the sample (VDL) was collected at a sand formation site. Small diversities and low numbers (10^2^–10^3^ cfu g^−1^ soil) of culturable actinobacteria have been recovered from each of these sampling sites^6, 55^.

### DNA extraction, PCR amplification and pyrosequencing

Total community DNA was extracted from all of the environmental samples using the proprietary UltraClean Soil DNA extraction kit (MoBIO Laboratories, Inc., USA) following the manufacturer’s protocol, and stored at −20 °C. Soil extractions were made in a laminar flow chamber using sterilized equipment in order to avoid contamination. The quality of community DNA preparations was checked by agarose gel electrophoresis and the sizes of the DNA fragments were compared with a molecular size marker (Gene Ruler^TM^, MBI Fermentas, Vilnius, Lithuania).

Actinobacterial specific regions in community DNA preparations were amplified using the primers Com2xf (5′-AAA CTC AAA GGA ATT GAC GG-3′) and Ac1186r (5′-CTT CCT CCG AGT TGA CCC-3′)^56^. Unique, sample-specific barcodes (10 bp) were attached to the 5′ end of the forward primer. Four replicates were constructed from each of the environmental samples, the latter were assigned individual barcodes.

PCR was made in 25 μl reaction mixtures containing 1 μl DNA template, 1x buffer (10x buffer: 160 mM (NH_4_)_2_SO_4_, 670 mMTris-HCl [pH 8.8 at 25 °C], 0.1% Tween 20), 0.125 mM each of the four dNTPs, 200 µM each of forward and reverse primers, 1.5 µM MgCl_2_ and 1.25 µM *Taq* polymerase. Positive and negative controls (*Verrucosispora maris* DSM 45365^T^ DNA and sterile distilled water, respectively) were incorporated into all PCR runs. The PCR reactions were carried out under the following conditions: initial denaturation at 94 °C for a minute, 30 cycles of 94 °C for a minute, 60 °C for 30 seconds and 72 °C for 30 seconds, and finally 72 °C for 5 minutes. The quality of the amplified products was established using agarose gel electrophoresis. The preparations were kept at −20 °C prior to use. PCR products were purified using the Agencourt AMPure XP PCR Purification System (Beckman Coulter, USA) and MagnaRack (Thermo Fisher Scientific Inc., USA) according to the manufacturers’ protocols. Sample concentrations were quantified using a Qubit® Fluorometer (Invitrogen, CA, USA) and adjusted to give a final concentration of 100 µg/mL; sample quality was checked using an Agilent Bioanalyzer by NewGene Ltd. (Newcastle upon Tyne, UK). Pyrosequencing was made using the Roche GS-FLX+ system (454 Life Sciences, Branford, CT) at the WM Keck Center for Comparative and Functional Genomics, University of Illinois, USA, as described in the manufacturer’s protocol. The library was sequenced at designated regions of a 70 × 75 PicoTiter Plate using flow pattern A with the Roche XL+ sequencing kit (454 Life Sciences) and software version 2.9. Signal processing and base calling were performed using the bundled 454 Data Analysis Software version 2.9 for amplicons.

### Bioinformatic analyses

High quality reads were processed using CLCommunity v3.30 software (www.chunlab.com) at ChunLab Inc., Seoul National University, Seoul, Korea). Processing steps involved trimming barcodes, filtering of low quality (<300 bp) and chimera sequences, taxonomic assignment, and statistical calculations using the ChunLab bioinformatics pipeline. The average post-processing sequence length was 283 bp. Taxonomic assignments of the sequence reads were made using the EzTaxon-e database^57^ (http://www.ezbiocloud.net/eztaxon) and a robust global pairwise sequencing alignment coupled with the BLASTN search tool (CLCommunity^TM^ User Manual, 2015). The similarity ranges used to identify operational taxonomic units (OTUs) were genus: 97-94.5 and family: 94.5-86.5 and taxonomic nomenclature was based on the list of prokaryotic names with standing in nomenclature (http://www.bacterio.net/). Chimera sequences formed during the PCR step were detected and removed from the dataset by UCHIME^58^. Sequences that could not be related to either validly published genera or families were given an accession number and an underscore suffix (e.g. family FJ479147_f, genus AM991247_g).

Rarefaction and rank-abundance analyses, Shannon diversities and Chao1 richness estimations were calculated using the CLCommunity software. OTU tables were saved in comma delimited (.csv) format prior to further analysis. Identified genera were checked and identified manually from the OTU table and analyzed to determine their composition in each of the environmental samples. Venn diagrams were constructed using the Mothur version 1.37.4 program (http://www.mothur.org/).

### Data availability statement

Comma separated value (csv) format data files and R scripts used in this study are available at https://github.com/rasanderson/sci_reports.

## Electronic supplementary material

Supplementary Tables
