# Supplementary material for: Actinobacterial Rare Biospheres and Dark Matter Revealed in Habitats of the Chilean Atacama Desert
Source: Sci Rep. 2017 Aug 21;7:8373. doi: 10.1038/s41598-017-08937-4 (PMC5566421; doi:10.1038/s41598-017-08937-4)
Supplement: Supplementary file 1 — Supplementary Tables [file 41598_2017_8937_MOESM1_ESM.doc]

**Actinobacterial Rare Biospheres and Dark Matter Revealed in Habitats of the Chilean Atacama Desert**

**Hamidah Idris^1^, Michael Goodfellow^1^, Roy Sanderson^1*^, Juan A Asenjo^2^, Alan T Bull^3^**

^1^School of Biology, Ridley Building, Newcastle University, Newcastle upon Tyne NE1 7RU, United Kingdom [roy.sanderson@ncl.ac.uk](mailto:roy.sanderson@ncl.ac.uk) ; [m.goodfellow@ncl.ac.uk](mailto:m.goodfellow@ncl.ac.uk)

^2^Centre for Biotechnology and Bioengineering (CeBiB), Department of Chemical Engineering and Biotechnology, University of Chile, Beauchef 851, Santiago, Chile [juasenjo@ing.uchile.cl](mailto:juasenjo@ing.uchile.cl)

^3^School of Biosciences, University of Kent, Canterbury CT2 7NJ, Kent, United Kingdom [A.T.Bull@kent.ac.uk](mailto:A.T.Bull@kent.ac.uk)

**Supplementary Table S1. Total of validated and candidate families detected.**

| No. | Taxonomy | Name |
| --- | --- | --- |
|  | FJ478799_c;;FJ478799_o;;FJ479147_f | FJ479147_f |
|  | *Acidimicrobia;;Acidimicrobiales;;Acidimicrobiaceae* | *Acidimicrobiaceae* |
|  | *Actinobacteria_c;;Frankiales;;Geodermatophilaceae* | *Geodermatophilaceae* |
|  | *Actinobacteria_c;;Micrococcales;;Microbacteriaceae* | *Microbacteriaceae* |
|  | *Actinobacteria_c;;Micrococcales;;Micrococcaceae* | *Micrococcaceae* |
|  | *Actinobacteria_c;;Frankiales;;*HQ910322_f | HQ910322_f |
|  | *Actinobacteria_c;;Micromonosporales;;Micromonosporaceae* | *Micromonosporaceae* |
|  | *Actinobacteria_c;;Propionibacteriales;;Nocardioidaceae* | *Nocardioidaceae* |
|  | *Acidimicrobia;;Acidimicrobiales;;Iamiaceae* | *Iamiaceae* |
|  | *Actinobacteria_c;;Corynebacteriales;;Nocardiaceae* | *Nocardiaceae* |
|  | *Actinobacteria_c;;Pseudonocardiales;;Pseudonocardiaceae* | *Pseudonocardiaceae* |
|  | *Actinobacteria_c;;Propionibacteriales;;Propionibacteriaceae* | *Propionibacteriaceae* |
|  | *Actinobacteria_c;;Frankiales;;Sporichthyaceae* | *Sporichthyaceae* |
|  | *Actinobacteria_c;;Streptomycetales;;Streptomycetaceae* | *Streptomycetaceae* |
|  | *Actinobacteria_c;;Micrococcales;;Sanguibacteraceae* | *Sanguibacteraceae* |
|  | *Actinobacteria_c;;Corynebacteriales;;Corynebacteriaceae* | *Corynebacteriaceae* |
|  | *Acidimicrobia;;Acidimicrobiales;;*AM991247_f | AM991247_f |
|  | *Actinobacteria_c;;Corynebacteriales;;Mycobacteriaceae* | *Mycobacteriaceae* |
|  | *Actinobacteria_c;;Micrococcales;;Intrasporangiaceae* | *Intrasporangiaceae* |
|  | *Actinobacteria_c;;Micrococcales;;Cellulomonadaceae* | *Cellulomonadaceae* |
|  | *Actinobacteria_c;;Kineosporiales;;Kineosporiaceae* | *Kineosporiaceae* |
|  | *Actinobacteria_c;;Frankiales;;Frankiaceae* | *Frankiaceae* |
|  | *Acidimicrobia;;Acidimicrobiales;;Ilumatobacter_f* | *Ilumatobacter_f* |
|  | *Actinobacteria_c;;Streptosporangiales;;Streptosporangiaceae* | *Streptosporangiaceae* |
|  | *Actinobacteria_c;;Corynebacteriales;;Dietziaceae* | *Dietziaceae* |
|  | *Actinobacteria_c;;Micrococcales;;Bogoriellaceae* | *Bogoriellaceae* |
|  | *Actinobacteria_c;;Frankiales;;*EU335288_f | EU335288_f |
|  | *Actinobacteria_c;;EF016800_o;;*EF016800_f | EF016800_f |
|  | *Actinobacteria_c;;Micrococcales;;Promicromonosporaceae* | *Promicromonosporaceae* |
|  | *Actinobacteria_c;;Jiangellales;;Jiangellaceae* | *Jiangellaceae* |
|  | *Actinobacteria_c;;Streptosporangiales;;Thermomonosporaceae* | *Thermomonosporaceae* |
|  | *Actinobacteria_c;;Streptosporangiales;;*AF498716_f | AF498716_f |
|  | *Actinobacteria_c;;Frankiales;;*EU861909_f | EU861909_f |
|  | *Acidimicrobiia;;Acidimicrobiales;;*DQ395423_f | DQ395423_f |
|  | *Actinobacteria_c;;Corynebacteriales;;*EF451703_f | EF451703_f |
|  | *Acidimicrobia;;Acidimicrobiales;;Microthrix_f* | *Microthrix_f* |
|  | *Actinobacteria_c;;Micrococcales;;Demequinaceae* | *Demequinaceae* |
|  | *Actinobacteria_c;;Actinomycetales;;Actinomycetaceae* | *Actinomycetaceae* |
|  | *Actinobacteria_c;;Micrococcales;;Dermabacteraceae* | *Dermabacteraceae* |
|  | *Actinobacteria_c;;Frankiales;;Cryptosporangiaceae* | *Cryptosporangiaceae* |
|  | *Actinobacteria_c;;Frankiales;;Nakamurellaceae* | *Nakamurellaceae* |
|  | *Actinobacteria_c;;Streptosporangiales;;Nocardiopsaceae* | *Nocardiopsaceae* |
|  | FJ478799_c;;FJ478799_o;;FJ478799_f | FJ478799_f |
|  | *Actinobacteria_c;;Micrococcales;;Brevibacteriaceae* | *Brevibacteriaceae* |
|  | *Acidimicrobia;;Acidimicrobiales;;*DQ396300_f | DQ396300_f |
|  | *Actinobacteria_c;;Micrococcales;;Rarobacteraceae* | *Rarobacteraceae* |
|  | *Acidimicrobia;;Acidimicrobiales;;*EU491192_f | EU491192_f |
|  | *Actinobacteria_c;;Micrococcales;;Dermatophilaceae* | *Dermatophilaceae* |
|  | *Acidimicrobia;;Acidimicrobiales;;*DQ395502_f | DQ395502_f |
|  | EU374107_c;;EU374107_o;;EU374107_f | EU374107_f |
|  | *Actinobacteria_c;;Frankiales;;*AB021325_f | AB021325_f |
|  | *Actinobacteria_c;;EF016806_o;;*EF016806_f | EF016806_f |
|  | *Actinobacteria_c;;Frankiales;;*AB245397_f | AB245397_f |
|  | *Actinobacteria_c;;Micrococcales;;Dermacoccaceae* | *Dermacoccaceae* |
|  | *Actinobacteria_c;;Micrococcales;;Beutenbergiaceae* | *Beutenbergiaceae* |
|  | EU374107_c;;EU374107_o;;EU374093_f | EU374093_f |
|  | *Acidimicrobia;;Acidimicrobiales;;*FN811204_f | FN811204_f |
|  | *Actinobacteria_c;;Motilibacter_o;;Motilibacteraceae* | *Motilibacteraceae* |
|  | *Actinobacteria_c;;Glycomycetales;;Glycomycetaceae* | *Glycomycetaceae* |
|  | *Actinobacteria_c;;Micrococcales;;Ruaniaceae* | *Ruaniaceae* |
|  | *Acidimicrobia;;Acidimicrobiales;;*DQ129383_f | DQ129383_f |
|  | *Rubrobacteria;;Gaiellales;;Gaiellaceae* | *Gaiellaceae* |
|  | *Actinobacteria_c;;Planktophila_o;;Planktophila_f* | *Planktophila_f* |
|  | *Actinobacteria_c;;Catenulisporales;;Catenulisporaceae* | *Catenulisporaceae* |
|  | *Actinobacteria_c;;Corynebacteriales;;Tsukamurellaceae* | *Tsukamurellaceae* |
|  | *Actinobacteria_c;;Catenulisporales;;Actinospicaceae* | *Actinospicaceae* |
|  | *Nitriliruptoria;;Nitriliruptorales;;Nitriliruptoraceae* | *Nitriliruptoraceae* |

Supplementary Table S2. Total of validated and candidate genera detected.

| **No.** | **Taxonomy** | **Name** |
| --- | --- | --- |
| 1 | FJ478799_c;;FJ478799_o;;FJ479147_f;;FJ479147_g | FJ479147_g |
| 2 | *Acidimicrobiia;;Acidimicrobiales;;Acidimicrobiaceae;;*HQ674860_g | HQ674860_g |
| 3 | *Actinobacteria_c;;Frankiales;;*HQ910322_f;;HQ910322_g | HQ910322_g |
| 4 | *Actinobacteria_c;;Frankiales;;Geodermatophilaceae;;Blastococcus* | *Blastococcus* |
| 5 | *Actinobacteria_c;;Micrococcales;;Micrococcaceae;;Arthrobacter* | *Arthrobacter* |
| 6 | *Actinobacteria_c;;Micromonosporales;;Micromonosporaceae;;Verrucosispora* | *Verrucosispora* |
| 7 | *Actinobacteria_c;;Frankiales;;Geodermatophilaceae;;Geodermatophilus* | *Geodermatophilus* |
| 8 | *Actinobacteria_c;;Frankiales;;Geodermatophilaceae;;Modestobacter* | *Modestobacter* |
| 9 | *Actinobacteria_c;;Corynebacteriales;;Nocardiaceae;;Gordonia* | *Gordonia* |
| 10 | *Actinobacteria_c;;Micrococcales;;Microbacteriaceae;;Microbacterium* | *Microbacterium* |
| 11 | *Actinobacteria_c;;Streptomycetales;;Streptomycetaceae;;Streptomyces* | *Streptomyces* |
| 12 | *Actinobacteria_c;;Frankiales;;Sporichthyaceae;;Sporichthya* | *Sporichthya* |
| 13 | *Actinobacteria_c;;Propionibacteriales;;Propionibacteriaceae;;Friedmanniella* | *Friedmanniella* |
| 14 | *Actinobacteria_c;;Micrococcales;;Micrococcaceae;;Kocuria* | *Kocuria* |
| 15 | *Actinobacteria_c;;Propionibacteriales;;Nocardioidaceae;;Nocardioides* | *Nocardioides* |
| 16 | *Actinobacteria_c;;Micrococcales;;Sanguibacteraceae;;Sanguibacter* | *Sanguibacter* |
| 17 | *Acidimicrobiia;;Acidimicrobiales;;Iamiaceae;;*HQ864103_g | HQ864103_g |
| 18 | *Actinobacteria_c;;Corynebacteriales;;Corynebacteriaceae;;Corynebacterium* | *Corynebacterium* |
| 19 | *Acidimicrobiia;;Acidimicrobiales;;Acidimicrobiaceae;;Aciditerrimonas* | *Aciditerrimonas* |
| 20 | *Acidimicrobiia;;Acidimicrobiales;;*AM991247_f;;FJ478790_g | FJ478790_g |
| 21 | *Actinobacteria_c;;Corynebacteriales;;Mycobacteriaceae;;Mycobacterium* | *Mycobacterium* |
| 22 | *Actinobacteria_c;;Micrococcales;;Microbacteriaceae;;Lysinimonas* | *Lysinimonas* |
| 23 | *Actinobacteria_c;;Pseudonocardiales;;Pseudonocardiaceae;;Pseudonocardia* | *Pseudonocardia* |
| 24 | *Actinobacteria_c;;Propionibacteriales;;Nocardioidaceae;;*HQ538692_g | HQ538692_g |
| 25 | *Actinobacteria_c;;Micrococcales;;Microbacteriaceae;;Amnibacterium* | *Amnibacterium* |
| 26 | *Actinobacteria_c;;Micrococcales;;Intrasporangiaceae;;Terrabacter* | *Terrabacter* |
| 27 | *Actinobacteria_c;;Pseudonocardiales;;Pseudonocardiaceae;;*JF266448_g | JF266448_g |
| 28 | *Acidimicrobiia;;Acidimicrobiales;;Iamiaceae;;*EF632905_g | EF632905_g |
| 29 | *Acidimicrobiia;;Acidimicrobiales;;Acidimicrobiaceae;;*EF516392_g | EF516392_g |
| 30 | *Acidimicrobiia;;Acidimicrobiales;;Acidimicrobiaceae;;*HQ190487_g | HQ190487_g |
| 31 | *Acidimicrobiia;;Acidimicrobiales;;Iamiaceae;;*EF018137_g | EF018137_g |
| 32 | *Actinobacteria_c;;Micrococcales;;Microbacteriaceae;;*EU861920_g | EU861920_g |
| 33 | *Actinobacteria_c;;Propionibacteriales;;Propionibacteriaceae;;Microlunatus* | *Microlunatus* |
| 34 | *Actinobacteria_c;;Micrococcales;;Microbacteriaceae;;Frigoribacterium* | *Frigoribacterium* |
| 35 | *Actinobacteria_c;;Micromonosporales;;Micromonosporaceae;;Actinoplanes* | *Actinoplanes* |
| 36 | *Actinobacteria_c;;Micrococcales;;Microbacteriaceae;;Diaminobutyricimonas* | *Diaminobutyricimonas* |
| 37 | *Actinobacteria_c;;Pseudonocardiales;;Pseudonocardiaceae;;Amycolatopsis* | *Amycolatopsis* |
| 38 | *Actinobacteria_c;;Corynebacteriales;;Nocardiaceae;;Nocardiaceae_uc* | *Nocardiaceae_*uc |
| 39 | *Actinobacteria_c;;Micrococcales;;Cellulomonadaceae;;Actinotalea* | *Actinotalea* |
| 40 | *Actinobacteria_c;;Frankiales;;Frankiaceae;;Jatrophihabitans* | *Jatrophihabitans* |
| 41 | *Actinobacteria_c;;Streptosporangiales;;Streptosporangiaceae;;Microbispora* | *Microbispora* |
| 42 | *Actinobacteria_c;;Micrococcales;;Cellulomonadaceae;;Cellulomonas* | *Cellulomonas* |
| 43 | *Actinobacteria_c;;Micrococcales;;Microbacteriaceae;;Naasia* | *Naasia* |
| 44 | *Actinobacteria_c;;Frankiales;;Sporichthyaceae;;*AF408985_g | AF408985_g |
| 45 | *Actinobacteria_c;;Corynebacteriales;;Dietziaceae;;Dietzia* | *Dietzia* |
| 46 | *Actinobacteria_c;;Propionibacteriales;;Nocardioidaceae;;*EU861822_g | EU861822_g |
| 47 | *Actinobacteria_c;;Micrococcales;;Microbacteriaceae;;Agromyces* | *Agromyces* |
| 48 | *Actinobacteria_c;;Kineosporiales;;Kineosporiaceae;;Kineococcus* | *Kineococcus* |
| 49 | *Acidimicrobiia;;Acidimicrobiales;;Ilumatobacter_f;;Ilumatobacter* | *Ilumatobacter* |
| 50 | *Actinobacteria_c;;Propionibacteriales;;Nocardioidaceae;;Marmoricola* | *Marmoricola* |
| 51 | *Actinobacteria_c;;Micromonosporales;;Micromonosporaceae;;Micromonospora* | *Micromonospora* |
| 52 | *Actinobacteria_c;;Micromonosporales;;Micromonosporaceae;;Couchioplanes* | *Couchioplanes* |
| 53 | *Actinobacteria_c;;Kineosporiales;;Kineosporiaceae;;*FM886842_g | FM886842_g |
| 54 | *Actinobacteria_c;;Micrococcales;;Bogoriellaceae;;Georgenia* | *Georgenia* |
| 55 | *Actinobacteria_c;;Frankiales;;*EU335288_f;;EU335288_g | EU335288_g |
| 56 | *Actinobacteria_c;;Propionibacteriales;;Propionibacteriaceae;;Propionibacterium* | *Propionibacterium* |
| 57 | *Actinobacteria_c;;*EF016800_o;;EF016800_f;;EF016800_g | EF016800_g |
| 58 | *Actinobacteria_c;;Propionibacteriales;;Nocardioidaceae;;Kribbella* | *Kribbella* |
| 59 | FJ478799_c;;FJ478799_o;;FJ479147_f;;FJ479147_f_uc | FJ479147_f_uc |
| 60 | *Actinobacteria_c;;Micrococcales;;Microbacteriaceae;;Frondihabitans* | *Frondihabitans* |
| 61 | *Actinobacteria_c;;Micromonosporales;;Micromonosporaceae;;Dactylosporangium* | *Dactylosporangium* |
| 62 | *Acidimicrobiia;;Acidimicrobiales;;Iamiaceae;;*JF319263_g | JF319263_g |
| 63 | *Actinobacteria_c;;Kineosporiales;;Kineosporiaceae;;Quadrisphaera* | *Quadrisphaera* |
| 64 | *Actinobacteria_c;;Micrococcales;;Micrococcaceae;;Micrococcus* | *Micrococcus* |
| 65 | *Acidimicrobiia;;Acidimicrobiales;;Acidimicrobiaceae;;*EF516593_g | EF516593_g |
| 66 | *Acidimicrobiia;;Acidimicrobiales;;Acidimicrobiaceae;;*FJ479034_g | FJ479034_g |
| 67 | *Actinobacteria_c;;Micrococcales;;Microbacteriaceae;;Homoserinimonas* | *Homoserinimonas* |
| 68 | *Actinobacteria_c;;Pseudonocardiales;;Pseudonocardiaceae;;Actinomycetospora* | *Actinomycetospora* |
| 69 | *Actinobacteria_c;;Propionibacteriales;;Nocardioidaceae;;Aeromicrobium* | *Aeromicrobium* |
| 70 | *Acidimicrobiia;;Acidimicrobiales;;Acidimicrobiaceae;;*EF127609_g | EF127609_g |
| 71 | *Actinobacteria_c;;Micrococcales;;Microbacteriaceae;;Herbiconiux* | *Herbiconiux* |
| 72 | *Actinobacteria_c;;Frankiales;;*EU861909_f;;EU861909_g | EU861909_g |
| 73 | *Acidimicrobiia;;Acidimicrobiales;;Acidimicrobiaceae;;Acidimicrobiaceae_uc* | *Acidimicrobiaceae*_uc |
| 74 | *Acidimicrobiia;;Acidimicrobiales;;*DQ395423_f;;DQ395423_g | DQ395423_g |
| 75 | *Actinobacteria_c;;Propionibacteriales;;Nocardioidaceae;;Flindersiella* | *Flindersiella* |
| 76 | *Actinobacteria_c;;Micrococcales;;Promicromonosporaceae;;Cellulosimicrobium* | *Cellulosimicrobium* |
| 77 | *Actinobacteria_c;;Micrococcales;;Demequinaceae;;Demequina* | *Demequina* |
| 78 | *Actinobacteria_c;;Pseudonocardiales;;Pseudonocardiaceae;;Lentzea* | *Lentzea* |
| 79 | *Actinobacteria_c;;Actinomycetales;;Actinomycetaceae;;Actinomyces* | *Actinomyces* |
| 80 | *Actinobacteria_c;;Kineosporiales;;Kineosporiaceae;;*EF127613_g | EF127613_g |
| 81 | *Acidimicrobiia;;Acidimicrobiales;;Iamiaceae;;*FM209170_g | FM209170_g |
| 82 | *Acidimicrobiia;;Acidimicrobiales;;Ilumatobacter_f;;*GQ387490_g | GQ387490_g |
| 83 | *Actinobacteria_c;;Propionibacteriales;;Nocardioidaceae;;Nocardioidaceae_uc* | *Nocardioidaceae*_uc |
| 84 | *Actinobacteria_c;;Corynebacteriales;;*EF451703_f;;EF451703_g | EF451703_g |
| 85 | *Actinobacteria_c;;Frankiales;;Frankiaceae;;*AY234742_g | AY234742_g |
| 86 | *Actinobacteria_c;;Streptosporangiales;;*AF498716_f;;EU861937_g | EU861937_g |
| 87 | *Acidimicrobiia;;Acidimicrobiales;;Iamiaceae;;Aquihabitans* | *Aquihabitans* |
| 88 | *Actinobacteria_c;;Jiangellales;;Jiangellaceae;;*EU735662_g | EU735662_g |
| 89 | *Actinobacteria_c;;Corynebacteriales;;Nocardiaceae;;Nocardia* | *Nocardia* |
| 90 | *Acidimicrobiia;;Acidimicrobiales;;Microthrix_f;;*EF516411_g | EF516411_g |
| 91 | *Actinobacteria_c;;Micromonosporales;;Micromonosporaceae;;Xiangella* | *Xiangella* |
| 92 | *Actinobacteria_c;;Micrococcales;;Dermabacteraceae;;Brachybacterium* | *Brachybacterium* |
| 93 | *Actinobacteria_c;;Micrococcales;;Microbacteriaceae;;*AY862797_g | AY862797_g |
| 94 | *Actinobacteria_c;;Micromonosporales;;Micromonosporaceae;;Pseudosporangium* | *Pseudosporangium* |
| 95 | *Actinobacteria_c;;Micromonosporales;;Micromonosporaceae;;Micromonosporaceae_uc* | *Micromonosporaceae_*uc |
| 96 | *Actinobacteria_c;;Pseudonocardiales;;Pseudonocardiaceae;;Pseudonocardiaceae_uc* | *Pseudonocardiaceae_*uc |
| 97 | *Actinobacteria_c;;Micrococcales;;Micrococcaceae;;Citricoccus* | *Citricoccus* |
| 98 | *Actinobacteria_c;;Micrococcales;;Microbacteriaceae;;Leifsonia* | *Leifsonia* |
| 99 | *Actinobacteria_c;;Frankiales;;Geodermatophilaceae;;Geodermatophilaceae_uc* | *Geodermatophilaceae_*uc |
| 100 | *Actinobacteria_c;;Kineosporiales;;Kineosporiaceae;;Angustibacter* | *Angustibacter* |
| 101 | *Acidimicrobiia;;Acidimicrobiales;;Iamiaceae;;*EU753662_g | EU753662_g |
| 102 | *Actinobacteria_c;;Micromonosporales;;Micromonosporaceae;;Phytohabitans* | *Phytohabitans* |
| 103 | *Actinobacteria_c;;Micrococcales;;Microbacteriaceae;;Agrococcus* | *Agrococcus* |
| 104 | *Actinobacteria_c;;Micrococcales;;Microbacteriaceae;;Cryobacterium* | *Cryobacterium* |
| 105 | *Actinobacteria_c;;Streptosporangiales;;Thermomonosporaceae;;Actinomadura* | *Actinomadura* |
| 106 | *Actinobacteria_c;;Micromonosporales;;Micromonosporaceae;;Catellatospora* | *Catellatospora* |
| 107 | *Actinobacteria_c;;Micrococcales;;Intrasporangiaceae;;Tetrasphaera* | *Tetrasphaera* |
| 108 | *Actinobacteria_c;;Streptosporangiales;;Thermomonosporaceae;;Actinoallomurus* | *Actinoallomurus* |
| 109 | *Actinobacteria_c;;Micrococcales;;Promicromonosporaceae;;Promicromonospora* | *Promicromonospora* |
| 110 | *Actinobacteria_c;;Micrococcales;;Intrasporangiaceae;;Phycicoccus* | *Phycicoccus* |
| 111 | *Actinobacteria_c;;Micrococcales;;Microbacteriaceae;;Pontimonas* | *Pontimonas* |
| 112 | *Actinobacteria_c;;Frankiales;;Cryptosporangiaceae;;*GQ088405_g | GQ088405_g |
| 113 | *Actinobacteria_c;;Frankiales;;Sporichthyaceae;;Sporichthyaceae_uc* | *Sporichthyaceae_*uc |
| 114 | *Actinobacteria_c;;Micrococcales;;Intrasporangiaceae;;Oryzihumus* | *Oryzihumus* |
| 115 | *Actinobacteria_c;;Jiangellales;;Jiangellaceae;;Jiangella* | *Jiangella* |
| 116 | *Actinobacteria_c;;Frankiales;;Frankiaceae;;Frankiaceae_uc* | *Frankiaceae_*uc |
| 117 | *Acidimicrobiia;;Acidimicrobiales;;Ilumatobacter_f;;*GQ487899_g | GQ487899_g |
| 118 | *Actinobacteria_c;;Pseudonocardiales;;Pseudonocardiaceae;;Lechevalieria* | *Lechevalieria* |
| 119 | *Actinobacteria_c;;Pseudonocardiales;;Pseudonocardiaceae;;Actinophytocola* | *Actinophytocola* |
| 120 | *Actinobacteria_c;;Micromonosporales;;Micromonosporaceae;;Virgisporangium* | *Virgisporangium* |
| 121 | *Actinobacteria_c;;Frankiales;;Sporichthyaceae;;*DQ413131_g | DQ413131_g |
| 122 | *Actinobacteria_c;;Micromonosporales;;Micromonosporaceae;;Catenuloplanes* | *Catenuloplanes* |
| 123 | *Actinobacteria_c;;Micrococcales;;Microbacteriaceae;;Curtobacterium* | *Curtobacterium* |
| 124 | *Actinobacteria_c;;Micrococcales;;Intrasporangiaceae;;Arsenicicoccus* | *Arsenicicoccus* |
| 125 | *Actinobacteria_c;;Frankiales;;*HQ910322_f;;AY250885_g | AY250885_g |
| 126 | *Actinobacteria_c;;Micrococcales;;Brevibacteriaceae;;Brevibacterium* | *Brevibacterium* |
| 127 | *Acidimicrobiia;;Acidimicrobiales;;Acidimicrobiaceae;;*GQ402597_g | GQ402597_g |
| 128 | *Actinobacteria_c;;Streptosporangiales;;Nocardiopsaceae;;Nocardiopsis* | *Nocardiopsis* |
| 129 | *Actinobacteria_c;;Corynebacteriales;;Nocardiaceae;;Rhodococcus* | *Rhodococcus* |
| 130 | *Actinobacteria_c;;Micromonosporales;;Micromonosporaceae;;Planosporangium* | *Planosporangium* |
| 131 | FJ478799_c;;FJ478799_o;;FJ478799_f;;EU132929_g | EU132929_g |
| 132 | *Actinobacteria_c;;Propionibacteriales;;Nocardioidaceae;;*EU289436_g | EU289436_g |
| 133 | *Acidimicrobiia;;Acidimicrobiales;;Ilumatobacter_f;;*AY093455_g | AY093455_g |
| 134 | *Acidimicrobiia;;Acidimicrobiales;;*EU491192_f;;EU491192_g | EU491192_g |
| 135 | *Actinobacteria_c;;Micrococcales;;Intrasporangiaceae;;Janibacter* | *Janibacter* |
| 136 | *Actinobacteria_c;;Frankiales;;*HQ910322_f;;HQ910322_f_uc | HQ910322_f_uc |
| 137 | *Actinobacteria_c;;Frankiales;;Frankiaceae;;Frankia* | *Frankia* |
| 138 | *Actinobacteria_c;;Kineosporiales;;Kineosporiaceae;;Kineosporiaceae_uc* | *Kineosporiaceae_*uc |
| 139 | *Actinobacteria_c;;Propionibacteriales;;Nocardioidaceae;;Thermasporomyces* | *Thermasporomyces* |
| 140 | *Acidimicrobiia;;Acidimicrobiales;;Acidimicrobiaceae;;*EF016795_g | EF016795_g |
| 141 | *Actinobacteria_c;;Micrococcales;;Dermatophilaceae;;Piscicoccus* | *Piscicoccus* |
| 142 | *Actinobacteria_c;;Corynebacteriales;;*EF451703_f;;EF451703_f_uc | EF451703_f_uc |
| 143 | *Acidimicrobiia;;Acidimicrobiales;;Iamiaceae;;*GQ487905_g | GQ487905_g |
| 144 | *Actinobacteria_c;;Micrococcales;;Microbacteriaceae;;Marisediminicola* | *Marisediminicola* |
| 145 | *Actinobacteria_c;;Frankiales;;Nakamurellaceae;;Nakamurella* | *Nakamurella* |
| 146 | *Actinobacteria_c;;Micrococcales;;Rarobacteraceae;;Rarobacter* | *Rarobacter* |
| 147 | *Acidimicrobiia;;Acidimicrobiales;;*AM991247_f;;AM991247_g | AM991247_g |
| 148 | *Actinobacteria_c;;Micrococcales;;Micrococcaceae;;Micrococcaceae_uc* | *Micrococcaceae_uc* |
| 149 | *Actinobacteria_c;;Micrococcales;;Sanguibacteraceae;;Sediminihabitans* | *Sediminihabitans* |
| 150 | *Actinobacteria_c;;Micromonosporales;;Micromonosporaceae;;Hamadaea* | *Hamadaea* |
| 151 | *Acidimicrobiia;;Acidimicrobiales;;Ilumatobacter_f;;*AJ863196_g | AJ863196_g |
| 152 | *Actinobacteria_c;;Micrococcales;;Intrasporangiaceae;;Ornithinicoccus* | *Ornithinicoccus* |
| 153 | *Actinobacteria_c;;Streptosporangiales;;*AF498716_f;;AF498716_g | AF498716_g |
| 154 | *Actinobacteria_c;;Micrococcales;;Intrasporangiaceae;;Ornithinimicrobium* | *Ornithinimicrobium* |
| 155 | *Actinobacteria_c;;Micromonosporales;;Micromonosporaceae;;Polymorphospora* | *Polymorphospora* |
| 156 | *Acidimicrobiia;;Acidimicrobiales;;*AM991247_f;;AM991247_f_uc | AM991247_f_uc |
| 157 | *Actinobacteria_c;;Micromonosporales;;Micromonosporaceae;;Rugosimonospora* | *Rugosimonospora* |
| 158 | *Acidimicrobiia;;Acidimicrobiales;;Microthrix_f;;*FN554394_g | FN554394_g |
| 159 | *Actinobacteria_c;;Jiangellales;;Jiangellaceae;;Jiangellaceae_uc* | *Jiangellaceae_*uc |
| 160 | *Actinobacteria_c;;Streptomycetales;;Streptomycetaceae;;Streptomycetaceae_uc* | *Streptomycetaceae_*uc |
| 161 | *Bacteria;;;Actinobacteria;;Acidimicrobiia;;Acidimicrobiales;;*DQ396300_f;;EF076187_g | EF076187_g |
| 162 | *Acidimicrobiia;;Acidimicrobiales;;Iamiaceae;;Iamiaceae_uc* | *Iamiaceae_*uc |
| 163 | *Actinobacteria_c;;Frankiales;;Nakamurellaceae;;*FM874283_g | FM874283_g |
| 164 | *Actinobacteria_c;;Micrococcales;;Microbacteriaceae;;Plantibacter* | *Plantibacter* |
| 165 | *Actinobacteria_c;;Micrococcales;;Micrococcaceae;;Auritidibacter* | *Auritidibacter* |
| 166 | *Acidimicrobiia;;Acidimicrobiales;;*DQ395502_f;;DQ395502_g | DQ395502_g |
| 167 | EU374107_c;;EU374107_o;;EU374107_f;;FJ712481_g | FJ712481_g |
| 168 | *Actinobacteria_c;;Pseudonocardiales;;Pseudonocardiaceae;;Saccharothrix* | *Saccharothrix* |
| 169 | *Actinobacteria_c;;Pseudonocardiales;;Pseudonocardiaceae;;Labedaea* | *Labedaea* |
| 170 | *Actinobacteria_c;;Frankiales;;Cryptosporangiaceae;;Cryptosporangium* | *Cryptosporangium* |
| 171 | *Actinobacteria_c;;Micromonosporales;;Micromonosporaceae;;Catelliglobosispora* | *Catelliglobosispora* |
| 172 | *Actinobacteria_c;;Micrococcales;;Microbacteriaceae;;Rathayibacter* | *Rathayibacter* |
| 173 | *Actinobacteria_c;;Pseudonocardiales;;Pseudonocardiaceae;;Actinosynnema* | *Actinosynnema* |
| 174 | *Acidimicrobiia;;Acidimicrobiales;;Acidimicrobiaceae;;*GU168000_g | GU168000_g |
| 175 | *Acidimicrobiia;;Acidimicrobiales;;Iamiaceae;;*FM209069_g | FM209069_g |
| 176 | *Actinobacteria_c;;*EF016806_o;;EF016806_f;;EF016806_g | EF016806_g |
| 177 | *Actinobacteria_c;;Micromonosporales;;Micromonosporaceae;;Spirilliplanes* | *Spirilliplanes* |
| 178 | *Actinobacteria_c;;Streptosporangiales;;*AF498716_f;;AF498716_f_uc | AF498716_f_uc |
| 179 | *Actinobacteria_c;;Micrococcales;;Micrococcaceae;;Rothia* | *Rothia* |
| 180 | *Actinobacteria_c;;Frankiales;;*AB245397_f;;AB245397_g | AB245397_g |
| 181 | *Actinobacteria_c;;Micrococcales;;Promicromonosporaceae;;Luteimicrobium* | *Luteimicrobium* |
| 182 | *Actinobacteria_c;;Streptosporangiales;;Streptosporangiaceae;;Nonomuraea* | *Nonomuraea* |
| 183 | *Actinobacteria_c;;Pseudonocardiales;;Pseudonocardiaceae;;*FJ379331_g | FJ379331_g |
| 184 | *Actinobacteria_c;;Micrococcales;;Microbacteriaceae;;Microbacteriaceae_uc* | *Microbacteriaceae_*uc |
| 185 | *Actinobacteria_c;;Micromonosporales;;Micromonosporaceae;;Jishengella* | *Jishengella* |
| 186 | *Actinobacteria_c;;Frankiales;;*AB021325_f;;FN687458_g | FN687458_g |
| 187 | *Actinobacteria_c;;Propionibacteriales;;Propionibacteriaceae;;Naumannella* | *Naumannella* |
| 188 | *Actinobacteria_c;;Micromonosporales;;Micromonosporaceae;;Luedemannella* | *Luedemannella* |
| 189 | *Actinobacteria_c;;Micrococcales;;Microbacteriaceae;;Subtercola* | *Subtercola* |
| 190 | *Actinobacteria_c;;Pseudonocardiales;;Pseudonocardiaceae;;Crossiella* | *Crossiella* |
| 191 | *Actinobacteria_c;;Micrococcales;;Microbacteriaceae;;*EF423344_g | EF423344_g |
| 192 | *Actinobacteria_c;;Propionibacteriales;;Propionibacteriaceae;;Propionibacteriaceae_uc* | *Propionibacteriaceae_*uc |
| 193 | EU374107_c;;EU374107_o;;EU374093_f;;EU374093_g | EU374093_g |
| 194 | *Actinobacteria_c;;Pseudonocardiales;;Pseudonocardiaceae;;Thermotunica* | *Thermotunica* |
| 195 | *Actinobacteria_c;;Micrococcales;;Micrococcaceae;;Nesterenkonia* | *Nesterenkonia* |
| 196 | *Acidimicrobiia;;Acidimicrobiales;;*DQ396300_f;;DQ396300_g | DQ396300_g |
| 197 | *Actinobacteria_c;;Corynebacteriales;;Corynebacteriaceae;;Corynebacteriaceae_uc* | *Corynebacteriaceae_*uc |
| 198 | *Actinobacteria_c;;Pseudonocardiales;;Pseudonocardiaceae;;Kibdelosporangium* | *Kibdelosporangium* |
| 199 | *Actinobacteria_c;;Micrococcales;;Microbacteriaceae;;Pseudoclavibacter* | *Pseudoclavibacter* |
| 200 | *Actinobacteria_c;;Propionibacteriales;;Nocardioidaceae;;Actinopolymorpha* | *Actinopolymorpha* |
| 201 | *Acidimicrobiia;;Acidimicrobiales;;*FN811204_f;;FN811204_g | FN811204_g |
| 202 | *Actinobacteria_c;;Micrococcales;;Intrasporangiaceae;;Knoellia* | *Knoellia* |
| 203 | *Actinobacteria_c;;Pseudonocardiales;;Pseudonocardiaceae;;Alloactinosynnema* | *Alloactinosynnema* |
| 204 | *Actinobacteria_c;;Micrococcales;;Microbacteriaceae;;Rudaibacter* | *Rudaibacter* |
| 205 | *Actinobacteria_c;;Motilibacter_o;;Motilibacteraceae;;Motilibacter* | *Motilibacter* |
| 206 | *Actinobacteria_c;;Jiangellales;;Jiangellaceae;;*JF727732_g | JF727732_g |
| 207 | *Actinobacteria_c;;Frankiales;;*AB021325_f;;AB021325_g | AB021325_g |
| 208 | *Actinobacteria_c;;Micrococcales;;Dermacoccaceae;;Luteipulveratus* | *Luteipulveratus* |
| 209 | *Acidimicrobiia;;Acidimicrobiales;;*DQ395502_f;;FJ229917_g | FJ229917_g |
| 210 | *Actinobacteria_c;;Micrococcales;;Microbacteriaceae;;Aquiluna* | *Aquiluna* |
| 211 | *Actinobacteria_c;;Kineosporiales;;Kineosporiaceae;;Kineosporia* | *Kineosporia* |
| 212 | *Actinobacteria_c;;Frankiales;;Cryptosporangiaceae;;Cryptosporangiaceae_uc* | *Cryptosporangiaceae_*uc |
| 213 | *Acidimicrobiia;;Acidimicrobiales;;*DQ395423_f;;DQ269060_g | DQ269060_g |
| 214 | *Actinobacteria_c;;Micrococcales;;Sanguibacteraceae;;Oerskovia* | *Oerskovia* |
| 215 | *Actinobacteria_c;;Micromonosporales;;Micromonosporaceae;;Longispora* | *Longispora* |
| 216 | *Actinobacteria_c;;Micrococcales;;Rarobacteraceae;;Rarobacteraceae_uc* | *Rarobacteraceae_*uc |
| 217 | *Actinobacteria_c;;Micrococcales;;Beutenbergiaceae;;Serinibacter* | *Serinibacter* |
| 218 | EU374107_c;;EU374107_o;;EU374107_f;;EU374107_g | EU374107_g |
| 219 | *Actinobacteria_c;;Micrococcales;;Microbacteriaceae;;Okibacterium* | *Okibacterium* |
| 220 | *Actinobacteria_c;;Micrococcales;;Microbacteriaceae;;Salinibacterium* | *Salinibacterium* |
| 221 | *Actinobacteria_c;;Glycomycetales;;Glycomycetaceae;;Glycomyces* | *Glycomyces* |
| 222 | *Actinobacteria_c;;Micrococcales;;Microbacteriaceae;;Chryseoglobus* | *Chryseoglobus* |
| 223 | *Actinobacteria_c;;Frankiales;;Frankiaceae;;Acidothermus* | *Acidothermus* |
| 224 | *Actinobacteria_c;;Streptomycetales;;Streptomycetaceae;;Streptacidiphilus* | *Streptacidiphilus* |
| 225 | *Acidimicrobiia;;Acidimicrobiales;;Iamiaceae;;Iamia* | *Iamia* |
| 226 | *Actinobacteria_c;;Micrococcales;;Beutenbergiaceae;;Miniimonas* | *Miniimonas* |
| 227 | *Actinobacteria_c;;Frankiales;;Sporichthyaceae;;*EU644212_g | EU644212_g |
| 228 | *Actinobacteria_c;;Propionibacteriales;;Propionibacteriaceae;;*DQ532175_g | DQ532175_g |
| 229 | *Actinobacteria_c;;Pseudonocardiales;;Pseudonocardiaceae;;Saccharopolyspora* | *Saccharopolyspora* |
| 230 | AB240310_c;;AB240310_o;;AB240310_f;;AB240310_f_uc | AB240310_f_uc |
| 231 | *Actinobacteria_c;;Micromonosporales;;Micromonosporaceae;;Phytomonospora* | *Phytomonospora* |
| 232 | FJ478799_c;;FJ478799_o;;FJ478799_f;;FJ478799_f_uc | FJ478799_f_uc |
| 233 | *Acidimicrobiia;;Acidimicrobiales;;Microthrix_f;;Microthrix* | *Microthrix* |
| 234 | *Actinobacteria_c;;Corynebacteriales;;Mycobacteriaceae;;Mycobacteriaceae_uc* | *Mycobacteriaceae_*uc |
| 235 | *Actinobacteria_c;;Micrococcales;;Microbacteriaceae;;Microcella* | *Microcella* |
| 236 | *Actinobacteria_c;;Micrococcales;;Promicromonosporaceae;;Promicromonosporaceae_uc* | *Promicromonosporaceae_*uc |
| 237 | *Actinobacteria_c;;Micrococcales;;Microbacteriaceae;;Labedella* | *Labedella* |
| 238 | *Acidimicrobiia;;Acidimicrobiales;;*DQ129383_f;;DQ129383_g | DQ129383_g |
| 239 | *Actinobacteria_c;;Micromonosporales;;Micromonosporaceae;;Actinocatenispora* | *Actinocatenispora* |
| 240 | *Actinobacteria_c;;Micromonosporales;;Micromonosporaceae;;Rhizocola* | *Rhizocola* |
| 241 | *Actinobacteria_c;;Frankiales;;*EU861909_f;;EU861909_f_uc | EU861909_f_uc |
| 242 | *Acidimicrobiia;;Acidimicrobiales;;Ilumatobacter_f;;*AJ863237_g | AJ863237_g |
| 243 | *Actinobacteria_c;;Micromonosporales;;Micromonosporaceae;;Allocatelliglobosispora* | *Allocatelliglobosispora* |
| 244 | *Actinobacteria_c;;Micrococcales;;Dermacoccaceae;;Dermacoccus* | *Dermacoccus* |
| 245 | *Acidimicrobiia;;Acidimicrobiales;;Microthrix_f;;Microthrix_f_uc* | *Microthrix_*f_uc |
| 246 | *Actinobacteria_c;;Streptosporangiales;;Thermomonosporaceae;;Spirillospora* | *Spirillospora* |
| 247 | *Actinobacteria_c;;Frankiales;;Cryptosporangiaceae;;Fodinicola* | *Fodinicola* |
| 248 | *Actinobacteria_c;;Streptosporangiales;;Thermomonosporaceae;;Actinocorallia* | *Actinocorallia* |
| 249 | *Actinobacteria_c;;Kineosporiales;;Kineosporiaceae;;Pseudokineococcus* | *Pseudokineococcus* |
| 250 | *Actinobacteria_c;;Pseudonocardiales;;Pseudonocardiaceae;;Kutzneria* | *Kutzneria* |
| 251 | *Actinobacteria_c;;Streptosporangiales;;Nocardiopsaceae;;Thermobifida* | *Thermobifida* |
| 252 | *Actinobacteria_c;;Micrococcales;;Ruaniaceae;;Ruania* | *Ruania* |
| 253 | *Actinobacteria_c;;Propionibacteriales;;Propionibacteriaceae;;Tessaracoccus* | *Tessaracoccus* |
| 254 | *Actinobacteria_c;;Micrococcales;;Cellulomonadaceae;;Cellulomonadaceae_uc* | *Cellulomonadaceae_*uc |
| 255 | *Actinobacteria_c;;Micrococcales;;Intrasporangiaceae;;Intrasporangiaceae_uc* | *Intrasporangiaceae_*uc |
| 256 | *Actinobacteria_c;;Pseudonocardiales;;Pseudonocardiaceae;;Saccharomonospora* | *Saccharomonospora* |
| 257 | *Nitriliruptoria;;Nitriliruptorales;;Nitriliruptoraceae;;Nitriliruptoraceae_uc* | *Nitriliruptoraceae_*uc |
| 258 | *Actinobacteria_c;;Micrococcales;;Intrasporangiaceae;;Intrasporangium* | *Intrasporangium* |
| 259 | *Actinobacteria_c;;Propionibacteriales;;Propionibacteriaceae;;Brooklawnia* | *Brooklawnia* |
| 260 | *Actinobacteria_c;;Micrococcales;;Intrasporangiaceae;;Humibacillus* | *Humibacillus* |
| 261 | *Actinobacteria_c;;Kineosporiales;;Kineosporiaceae;;*DQ532344_g | DQ532344_g |
| 262 | *Actinobacteria_c;;Micrococcales;;Sanguibacteraceae;;Sanguibacteraceae_uc* | *Sanguibacteraceae_*uc |
| 263 | *Actinobacteria_c;;Jiangellales;;Jiangellaceae;;Haloactinopolyspora* | *Haloactinopolyspora* |
| 264 | *Acidimicrobiia;;Acidimicrobiales;;*DQ396300_f;;DQ396300_f_uc | DQ396300_f_uc |
| 265 | *Actinobacteria_c;;Propionibacteriales;;Propionibacteriaceae;;Mariniluteicoccus* | *Mariniluteicoccus* |
| 266 | *Actinobacteria_c;;Micrococcales;;Promicromonosporaceae;;*EF157137_g | EF157137_g |
| 267 | *Actinobacteria_c;;Pseudonocardiales;;Pseudonocardiaceae;;Umezawaea* | *Umezawaea* |
| 269 | *Rubrobacteria;;Gaiellales;;Gaiellaceae;;Gaiellaceae_uc* | *Gaiellaceae_*uc |
| 270 | *Actinobacteria_c;;Corynebacteriales;;Dietziaceae;;Dietziaceae_uc* | *Dietziaceae_uc* |
| 271 | *Actinobacteria_c;;Corynebacteriales;;Tsukamurellaceae;;Tsukamurella* | *Tsukamurella* |
| 272 | *Actinobacteria_c;;Catenulisporales;;Catenulisporaceae;;Catenulispora* | *Catenulispora* |
| 273 | *Actinobacteria_c;;Micrococcales;;Microbacteriaceae;;Mycetocola* | *Mycetocola* |
| 274 | *Actinobacteria_c;;Micrococcales;;Microbacteriaceae;;Humibacter* | *Humibacter* |
| 275 | *Actinobacteria_c;;Frankiales;;*AB021325_f;;AB021325_f_uc | AB021325_f_uc |
| 276 | *Actinobacteria_c;;Micrococcales;;Microbacteriaceae;;Glaciibacter* | *Glaciibacter* |
| 277 | *Actinobacteria_c;;Micromonosporales;;Micromonosporaceae;;Plantactinospora* | *Plantactinospora* |
| 278 | *Actinobacteria_c;;Micromonosporales;;Micromonosporaceae;;Salinispora* | *Salinispora* |
| 279 | *Actinobacteria_c;;Pseudonocardiales;;Pseudonocardiaceae;;Actinokineospora* | *Actinokineospora* |
| 280 | *Actinobacteria_c;;Actinomycetales;;Actinomycetaceae;;Actinomycetaceae_uc* | *Actinomycetaceae_uc* |
| 281 | *Actinobacteria_c;;Pseudonocardiales;;Pseudonocardiaceae;;Allokutzneria* | *Allokutzneria* |
| 282 | *Actinobacteria_c;;Micrococcales;;Intrasporangiaceae;;*JN588609_g | JN588609_g |
| 283 | *Actinobacteria_c;;Frankiales;;Frankiaceae;;*EU132518_g | EU132518_g |
| 284 | *Actinobacteria_c;;Micrococcales;;Micrococcaceae;;Zhihengliuella* | *Zhihengliuella* |
| 285 | *Actinobacteria_c;;Micromonosporales;;Micromonosporaceae;;*FN667447_g | FN667447_g |
| 286 | *Actinobacteria_c;;Micrococcales;;Dermacoccaceae;;Rudaeicoccus* | *Rudaeicoccus* |
| 287 | *Actinobacteria_c;;Planktophila_o;;Planktophila_f;;Planktophila* | *Planktophila* |
| 288 | *Actinobacteria_c;;Streptomycetales;;Streptomycetaceae;;Kitasatospora* | *Kitasatospora* |
| 289 | *Actinobacteria_c;;Micrococcales;;Micrococcaceae;;Tersicoccus* | *Tersicoccus* |
| 290 | *Actinobacteria_c;;Corynebacteriales;;Mycobacteriaceae;;Hoyosella* | *Hoyosella* |
| 291 | *Actinobacteria_c;;Streptosporangiales;;Nocardiopsaceae;;Nocardiopsaceae_uc* | *Nocardiopsaceae_*uc |
| 292 | *Actinobacteria_c;;Micromonosporales;;Micromonosporaceae;;Asanoa* | *Asanoa* |
| 293 | *Actinobacteria_c;;Glycomycetales;;Glycomycetaceae;;Glycomycetaceae_uc* | *Glycomycetaceae_*uc |
| 294 | *Actinobacteria_c;;Corynebacteriales;;Corynebacteriaceae;;Turicella* | *Turicella* |
| 295 | *Actinobacteria_c;;Micrococcales;;Beutenbergiaceae;;Salana* | *Salana* |
| 296 | *Actinobacteria_c;;Catenulisporales;;Actinospicaceae;;Actinospica* | *Actinospica* |
| 297 | *Actinobacteria_c;;Micrococcales;;Promicromonosporaceae;;Myceligenerans* | *Myceligenerans* |
